# Predictors of asthma exacerbations in children with or without house dust mite allergy

**DOI:** 10.13075/ijomeh.1896.02867

**Published:** 2026

**Authors:** Katarzyna Gmachowska, Izabela Socha-Wojtylak, Paweł Majak, Joanna Jerzyńska

**Affiliations:** 1 Medical University of Lodz, Department of Pediatrics and Allergy, Łódź, Poland; 2 University of Lodz, Institute of Psychology, Łódź, Poland; 3 Medical University of Lodz, Department of Pediatric Pulmonology, Łódź, Poland

**Keywords:** asthma exacerbation, pediatric asthma, asthma markers, house dust mite allergy, soluble receptor for advanced glycation end products, sRAGE

## Abstract

**Objectives:**

Asthma exacerbations in children represent a significant contributor to disease burden, with the risk of occurrence depending on the type of allergic sensitization. Identification of clinical and biological factors may improve risk stratification and support therapeutic management. The aim of this study was to assess the relationships between exacerbation frequency, lung function, inflammatory biomarkers, and the severity of sinonasal symptoms in children with asthma.

**Material and Methods:**

In a retrospective observational study, 80 children with allergic rhinitis and asthma were enrolled. Patients were stratified according to exacerbation frequency (<2 vs. ≥2 over 12 months) and sensitization to house dust mites (HDM). Assessments included spirometry (forced expiratory volume in 1 s [FEV₁], FEV₁/forced vital capacity [FVC]), fractional exhaled nitric oxide (FeNO), peripheral blood eosinophil count, and AGE-RAGE (advanced glycation end-products-receptor for advanced glycation end-products) pathway activity assessed by skin autofluorescence (SAF). Asthma control was evaluated using the *Childhood Asthma Control Test* (c-ACT), while sinonasal symptoms were assessed using the *Sinus and Nasal Quality of Life Survey* (SN-5) questionnaire.

**Results:**

In children without sensitization to HDM, more frequent exacerbations correlated with higher sRAGE concentrations (p = 0.0259) and greater activity limitation due to sinonasal symptoms (p = 0.0309). In HDM-sensitized children, frequent exacerbations were associated with lower FEV₁ (p = 0.0089), reduced FEV₁/FVC (p = 0.0186), and poorer asthma control (p = 0.0117). Greater severity of sinonasal symptoms was observed in children with poorer asthma control (p = 0.012).

**Conclusions:**

Exacerbation risk in asthma is associated with distinct profiles depending on sensitization status. Elevated sRAGE concentrations and increased sinonasal inflammation may characterize phenotypes not associated with HDM. In contrast, impaired lung function and poor asthma control predominate in HDM-associated asthma.

## Highlights

- Higher *Sinus and Nasal Quality of Life Survey* scores associate with poorer asthma control in children.
- Sinonasal symptoms reflect risk of asthma exacerbations.
- Soluble receptor for advanced glycation end products (sRAGE) and fractional exhaled nitric oxide show complementary inflammatory patterns.
- Sinonasal symptoms may support asthma control assessment.
- The sRAGE was explored as a novel biomarker in pediatric asthma.

## INTRODUCTION

Asthma is one of the most common chronic respiratory conditions in children and remains a significant health problem worldwide. Despite continuous advances in the treatment and monitoring of asthma, its exacerbations remain a significant cause of reduced physical and social activity, increased frequency of visits to accident and emergency (A&E) departments and hospitalizations in the pediatric population, and even death. Preventing exacerbations is therefore one of the most important goals of asthma management, as these events are associated with substantial clinical burden and reduced quality of life [1,2].

Asthma is a heterogeneous disease that encompasses various clinical and inflammatory subtypes based on type 2 inflammation, as well as phenotypes independent of this mechanism. These differ in terms of pathophysiological mechanisms, biomarker profiles and response to treatment. Biomarkers used to assess airway inflammation, such as fractional exhaled nitric oxide (FeNO) and peripheral blood eosinophil count, primarily reflect type 2 in flammation. They do not fully capture the complexity of asthma's pathophysiology, particularly in children with non-allergic phenotypes [3].

Increasing attention is being paid to the signaling pathway of the receptor for advanced glycation end-products (RAGE). It is a multi-ligand receptor involved in inflammatory processes, oxidative stress and tissue damage, including in the lungs. The soluble form of this receptor for advanced glycation end-products (sRAGE) acts as a so-called decoy receptor, which is involved in binding pro-inflammatory ligands and modulating the inflammatory response. Dysfunction of the RAGE pathway occurs in many lung diseases, such as chronic obstructive pulmonary disease, idiopathic pulmonary fibrosis, acute lung injury, asthma and cystic fibrosis. However, the role of sRAGE in the pathogenesis and course of asthma in the pediatric population remains poorly understood [4,5].

Another significant factor influencing the course of asthma in children is the interaction between upper and lower respiratory tract conditions. A close anatomical and immunological relationship between the nasal mucosa and the bronchial mucosa has been described. Inflammation of the upper airways can lead to an exacerbation of symptoms in the lower airways and a loss of control over asthma [6,7].

However, determining the relationship between the severity of sinus and nasal symptoms, impaired lung function, and new biomarkers of inflammation, such as RAGE, in predicting asthma exacerbations in children requires further research.

The authors hypothesized that clinical, functional, and inflammatory markers may differ between children with frequent and infrequent asthma exacerbations and that these associations may vary according to house dust mites (HDM) sensitization status. The aim of this study was to evaluate associations between asthma exacerbation frequency, lung function parameters, inflammatory biomarkers (including sRAGE), and sinonasal symptom severity in children with asthma with and without confirmed HDM allergy.

## MATERIAL AND METHODS

The study included 80 children and adolescents aged 4–18 years with physician-diagnosed asthma ≤12 months prior to study inclusion. Participants were recruited either from an outpatient allergy clinic or during hospitalization due to an asthma exacerbation. The diagnosis of asthma was established in accordance with the international guidelines of the Global Initiative for Asthma (GINA) [1,8].

An asthma exacerbation was defined in accordance with the recommendations of GINA as an acute worsening of symptoms requiring treatment with systemic corticosteroids, an unscheduled medical consultation, a visit to the accident and emergency department, or hospitalization. The number of exacerbations that had occurred in the past 12 months was obtained from medical records and confirmed during a clinical interview with the patient and their caregivers [1,2,8].

Participants were stratified into 2 groups based on exacerbation frequency during the previous 12 months: children with <2 exacerbations annually (0–1 exacerbation) and children ≥2 exacerbations annually. Additionally, subgroup analyses were performed according to confirmed HDM sensitization status.

All participants were assessed during clinical stability, and study procedures were performed after resolution of acute exacerbation symptoms. Prior to inclusion in the study, written informed consent was obtained from parents or legal guardians, and additionally from participants aged ≥16 years.

Exclusion criteria included the presence of structural or functional abnormalities of the upper or lower airways, participant's age <4 years or >18 years, hypertrophy of the adenoidal tonsil meeting surgical criteria for adenotomy, long-term systemic corticosteroids, history of malignancy, chronic diseases affecting respiratory function (such as cystic fibrosis and diabetes), primary immunodeficiency disorders involving humoral or cellular immunity and inability to cooperate during the performed diagnostic procedures.

### Assessment of allergic status

Information regarding allergic status was obtained retrospectively from available medical records. Sensitization to HDM was confirmed based on previously documented positive skin prick tests and/or elevated serum allergen-specific immunoglobulin E (IgE) levels to common aeroallergens, including HDM. Information regarding concomitant allergic rhinitis was also collected from medical documentation.

### Measurement of NO concentration in exhaled air

During the study visit, lung function was assessed using spirometry and airway inflammation was evaluated by measuring FeNO. Exhaled nitric oxide levels were assessed in all participants prior to spirometry, as forced exhalations during spirometry may temporarily lower exhaled nitric oxide levels, which could compromise the accuracy of the measurement [1,8–10]. Participants continued their standard inhaled therapy according to routine clinical practice, which should be considered when interpreting FeNO results.

Fractional exhaled nitric oxide was measured using a nitric oxide analyzer (model 280i, Sievers Instruments Inc., Boulder, CO, USA), according to the recommendations of American Thoracic Society/European Respiratory Society (ATS/ERS) [8,11]. The FENO test was performed using the restricted exhaled breath REB technique at a constant expiratory flow rate of 50 ml/s. Three technically valid measurements were obtained for each participant, and the final FeNO value was the average of these measurements. Results were expressed in parts per billion (ppb).

### Pulmonary function testing

Resting spirometry with flow–volume curve analysis was performed using a MasterScreen device (Jaeger, Hochberg, Germany). All pulmonary function tests were conducted in the Respiratory Function Testing Laboratory under standardized conditions between 9:00 a.m. and 11:00 a.m. to minimize potential circadian variability. During the examination, participants perform forced expiratory maneuvers following maximal inspiration in accordance with standardized spirometric procedures. Measurements were carried out in accordance with the joint ATS/ERS guidelines on spirometry [8–11].

### Measurement of advanced glycation end products

Advanced glycation end products (AGEs) were indirectly quantified by measuring skin autofluorescence (SAF) using an AGE Reader (DiagnOptics BV, Groningen, The Netherlands). The device illuminates approx. 4 cm^2^ of the skin on the volar side of the forearm with a UV-A source (300–420 nm). The emitted fluorescence (420–600 nm) and reflected light are measured with a spectrometer. Skin autofluorescence is calculated as the ratio of the average emitted light intensity to the average reflected light intensity, expressed in arbitrary units (a.u.) [12,13]. All measurements were performed on skin areas free of visible abnormalities, creams, or sunscreens. This measurement was used as an indirect surrogate marker related to AGE-RAGE pathway activity.

### Peripheral blood eosinophil assessment

Peripheral blood eosinophil counts were assessed retrospectively based on data extracted from patients' medical records. The analysis was based on the most recent laboratory test results ordered by the attending physician at an outpatient allergy clinic or during hospitalization. This single-time-point assessment may not reflect longitudinal eosinophilic variability and represents a methodological limitation.

Asthma control was assessed using validated pediatric questionnaires, the *Childhood Asthma Control Test* (c-ACT). To measure longitudinal change in health-related quality of life for children with persistent sinonasal symptoms (aller gic rhinitis), *Sinus and Nasal Quality of Life Survey* (SN-5) was used [5,6].

The study protocol was approved by the Bioethics Committee of the Medical University of Łódź (approval No. RNN/104/21/KE; May 11, 2021). The study was funded by a research grant from the Polpharma Scientific Foundation (5/XIX/2020).

### Statistical analysis

All variables were compared between groups using Fisher's exact test for categorical variables and the Mann-Whitney U test for continuous variables. The Spearman rank correlation coefficient was used to assess the strength and direction of a relationship between two ranked variables. A p-value <0.05 was considered statistically significant. All analyses were performed using Statistica 13.1 (TIBCO Software Inc., Palo Alto, USA).

## RESULTS

A total of 80 children participated in the study. The baseline characteristics of the study population are summarized in Table 1.

**Table 1:** Baseline characteristics of 80 children aged 4–18 years with asthma enrolled at the Medical University of Lodz, Łódź, Poland, 2021–2024

Variable	Me	Q25	Q75	M	SD
Socioeconomic					
age [years]	11.0	9.0	14.0	11.0	3.2
BMI [kg/m^2^]	19.0	17.0	22.0	19.8	4.0
Medical					
FEV_1_ [%]	98.5	90.0	114.0	101.5	16.3
FEV_1_/FVC [%]	100.0	94.0	105.0	99.9	9.3
FeNO [ppb]	39.8	31.0	61.0	50.0	26.7
Eo [cell/mm^3^]	331.0	200.0	540.0	398.5	280.7
sRAGE	1.1	1.0	1.2	1.1	0.2
c-ACT [pts]	22.0	18.0	24.0	20.7	4.6
SN-5 total [pts]	3.0	2.4	3.9	3.2	1.2
sinus infection	3.0	3.0	4.0	3.5	1.5
nasal obstruction	4.0	3.0	4.0	3.7	1.5
allergy symptoms	3.0	2.0	4.0	3.5	1.5
emotional distress	3.0	2.0	4.0	2.9	1.7
activity limitations	3.0	2.0	4.0	2.9	1.6

BMI – body mass index; Eo – eosinophilia; FeNO – fractional exhaled nitric oxide; FEV_1_ – forced expiratory volume in 1 s; FEV_1_/FVC – forced expiratory volume in 1 s/forced vital capacity; sRAGE – soluble receptor for advanced glycation end-products.

c-ACT – *Childhood Asthma Control Test*; SN-5 – *Sinus and Nasal Quality of Life Survey*.

Comparative analyses between children experiencing <2 asthma exacerbations/year (0 or 1) (N = 38) and those with ≥2 exacerbations during the last 12-months (N = 42) are presented in Table 2. Analyses were stratified according to HDM sensitization status as predefined in the study design.

**Table 2:** Comparison of clinical characteristics between children with frequent (≥2/year) and infrequent (<2/year) asthma exacerbations among 80 children with asthma, Medical University of Lodz, Łódź, Poland, May 2021 – December 2024

Variable	Children with asthma exacerbations (N = 80)						
	<2/year (N = 38)			≥2/year (N = 42)			
	Me	Q25	Q75	Me	Q25	Q75	p
Children house dust mite negative (N = 36)							
socioeconomic							
age [years]	8.5	11.0	13.0	7.0	8.0	14.0	0.5682
BMI [kg/m^2^]	17.5	20.0	22.0	16.0	17.0	20.0	0.2128
medical							
FEV_1_ [%]	97.5	106.5	123.0	93.0	97.0	116.0	0.2798
FEV_1_/FVC [%]	95.5	100.5	107.0	99.0	102.0	113.0	0.5469
FeNO [ppb]	31.5	40.7	56.5	30.0	36.0	38.2	0.0858
Eo [cell/mm^3^]	145.0	321.0	492.0	200.0	230.0	430.0	0.8180
sRAGE	0.9	1.1	1.2	1.1	1.2	1.2	0.0259
c-ACT [pts]	20.0	23.0	24.0	16.0	18.0	24.0	0.1993
SN-5 total [pts]	2.8	3.0	4.0	2.8	4.4	4.6	0.1252
sinus infection	3.0	3.0	4.0	3.0	4.0	6.0	0.3285
nasal obstruction	2.0	4.0	4.0	3.0	5.0	5.0	0.2603
allergy symptoms	2.0	3.0	4.0	3.0	5.0	5.0	0.0539
emotional distress	2.0	3.0	4.0	1.0	2.0	5.0	0.6385
activity limitations	2.0	2.0	3.0	2.0	4.0	5.0	0.0309
Children house dust mite positive (N = 44)							
socioeconomic							
age [years]	9.0	10.5	14.0	10.0	11.0	12.0	0.7200
BMI [kg/m^2^]	17.0	19.0	21.5	17.0	19.5	23.0	0.7778
medical							
FEV_1_ [%]	90.0	98.0	110.5	76.0	87.0	97.0	0.0089
FEV_1_/FVC [%]	94.5	101.0	105.5	89.0	92.5	100.0	0.0186
FeNO [ppb]	30.7	38.3	66.0	37.2	45.0	74.0	0.4426
Eo [cell/mm^3^]	236.0	347.0	508.5	230.0	342.0	631.0	0.9294
sRAGE	1.1	1.2	1.2	1.1	1.1	1.2	0.6272
c-ACT [points]	19.5	22.5	24.0	15.0	19.0	20.0	0.0117
SN-5 total [pts]	2.0	3.0	3.8	2.4	3.2	4.8	0.5211
sinus infection	2.0	3.0	4.0	3.0	3.0	4.0	0.9301
nasal obstruction	3.0	4.0	4.0	3.0	4.0	5.0	0.3133
allergy symptoms	2.0	3.0	4.0	2.0	3.0	6.0	0.8619
emotional distress	1.0	3.0	3.0	1.0	3.0	4.0	0.8095
activity limitations	1.0	2.0	4.0	3.0	3.0	3.0	0.1740

Abbreviations as in Table 1.

Comparisons were carried out in separate subgroups of children with and without house dust mite allergy.

### Children without HDM allergy

In the group of children without HDM allergy (N = 36), most demographic and clinical parameters did not differ significantly between the groups with more frequent and less frequent exacerbations. Age, BMI, spirometric parameters, FeNO levels, eosinophil counts, and asthma control scores were comparable between groups (Table 2).

**Figure 1:**
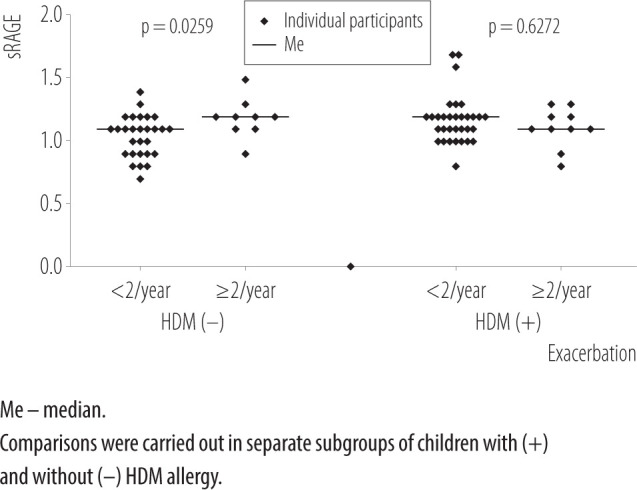
Soluble receptor for advanced glycation end-products (sRAGE) according to asthma exacerbation frequency in children with asthma who have experienced either more frequent or less frequent exacerbations in the past 12 months stratified by house dust mite (HDM) sensitization status, Medical University of Lodz, Łódź, Poland, May 2021 – December 2024

However, children with more frequent asthma exacerbations were found to have significantly AGE-RAGE pathway activity assessed by SAF compared to children with fewer exacerbations (p = 0.0259) (Table 2 and Figure 1).

Limitations in daily activities, as assessed using the SN-5 questionnaire, were significantly greater in children with frequent exacerbations (p = 0.0309) (Table 2).

### Children with HDM allergy

In contrast, among children allergic to HDM (N = 44), frequent exacerbations correlated with significantly poorer lung function parameters. In the group of children with ≥2 exacerbations/year, significantly lower FEV_1_ values (p = 0.0089) and lower FEV_1_/FVC ratios (p = 0.0186) were observed. This indicates greater airflow limitation. Asthma control was also significantly poorer in this group, as reflected by lower scores on the c-ACT scale (p = 0.0117) (Table 2).

No significant differences were observed between the groups of exacerbation frequency in terms of FeNO levels, eosinophil counts, SAF valuesor SN-5 scores.

### The relationship between nasal and sinus symptoms and asthma control

**Figure 2:**
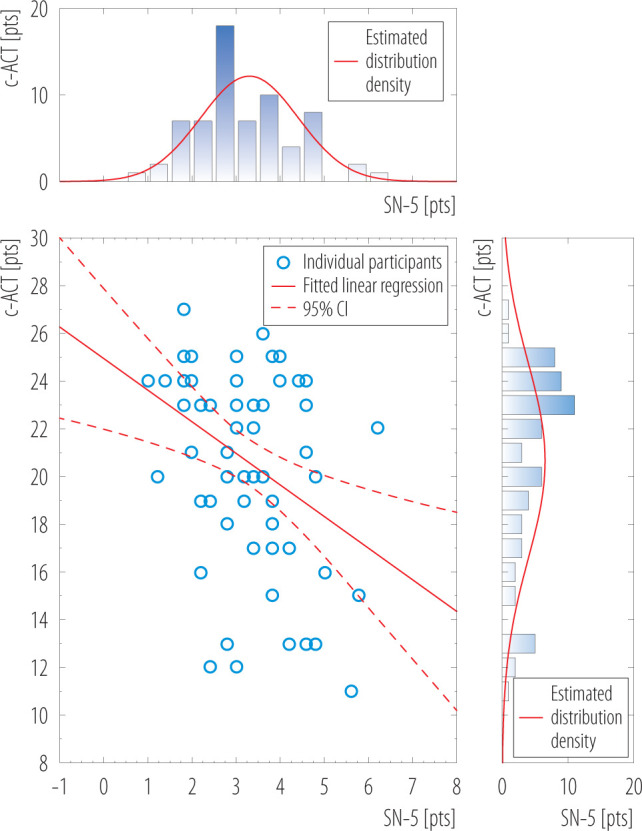
Correlation between *Sinus and Nasal Quality of Life Survey* (SN-5) and *Childhood Asthma Control Test* (c-ACT) scores in 80 children with asthma, Medical University of Lodz, Łódź, Poland, May 2021 – December 2024

The relationship between nasal and sinus symptoms due to allergic rhinitis, and asthma control is presented in Table 3 and Figure 2. A statistically significant correlation was demonstrated between SN-5 scores and c-ACT scores (p = 0.012). In children with lower SN-5 scores (<3.5), indicating milder sinonasal symptoms, good asthma control (c-ACT >19) was observed in 78% of cases. In contrast, among children with more severe sinonasal symptoms (SN-5 ≥3.5), good asthma control was observed in only 45% of cases.

**Table 3:** Association between *Sinus and Nasal Quality of Life Survey* (SN-5) and *Childhood Asthma Control Test* (c-ACT) scores in 80 children with asthma, Medical University of Lodz, Łódź, Poland, May 2021 – December 2024

c-ACT	Children with asthma (N = 80)			
	SN-5 <3.5 pts		SN-5 ≥3.5 pts	
	n	%	n	%
>19 pts	41	78	12	45
≤19 pts	12	22	15	55

p = 0.012.

Poor asthma control (c-ACT ≤19) was present in 55% of children with higher SN-5 scores, compared with 22% in those with lower SN-5 scores.

### The relationship between serum sRAGE concentrations and asthma exacerbation frequency

The relationship between serum sRAGE concentrations and asthma exacerbation frequency is illustrated in Figure 1. Higher sRAGE concentrations were observed in children without sensitization to HDM who experienced more frequent exacerbations. However, no such association was found in children with HDM allergy.

## DISCUSSION

Asthma exacerbations are a significant factor affecting children's quality of life and therefore pose a major challenge in the management of this condition [1,2]. Identifying clinical and biological markers that increase the risk of asthma exacerbations is therefore crucial for improving the early detection of loss of disease control and for implementing appropriate personalized treatment. This study assessed the association between the frequency of asthma exacerbations, lung function parameters, inflammatory biomarkers, and the severity of sinus and nasal symptoms in a group of children with asthma, with or without HDM allergy. Importantly, due to the cross-sectional design and retrospective assessment of prior exacerbations, the present findings should be interpreted as associations rather than causal relationships. The results suggest that factors influencing the risk of exacerbations may vary depending on the presence of allergen hypersensitivity. This highlights the heterogeneity of asthma phenotypes in the pediatric population and may suggests the involvement of distinct inflammatory pathways in allergic and non-allergic asthma.

In children allergic to HDM, more frequent exacerbations may be associated with reduced lung ventilation parameters and poorer asthma control. In particular children who experienced at least two exacerbations during the observation period had significantly lower FEV_1_ and FEV_1_/FVC values. These findings suggest that impaired lung function may be associated with higher exacerbation burden; however, causality cannot be established because lung function was assessed after exacerbation events had already occurred. These findings are consistent with previous studies suggesting that reduced lung ventilation parameters constitute an important prognostic factor for asthma exacerbations in children. They also reflect persistent airway inflammation and bronchial wall remodelling [1,8–10].

Interestingly, typical biomarkers of type 2 inflammation, including FeNO and peripheral blood eosinophil count, did not differ significantly between groups of children with frequent and infrequent exacerbations. Although FeNO is a commonly used non-invasive marker of eosinophilic airway inflammation, its predictive value for asthma exacerbations remains inconclusive in the pediatric population. Importantly, FeNO levels are strongly influenced by treatment with inhaled corticosteroids [14,15].

The results of our study indicate that, in some children with allergic asthma, physiological indicators – such as control of clinical symptoms and deterioration in lung function – may provide more reliable information regarding an increased risk of exacerbations than individual biomarkers of inflammation.

A different clinical picture was observed in children who were not allergic to HDM. In this subgroup, frequent exacerbations were associated with higher AGE-RAGE pathway activity assessed indirectly by SAF, as well as greater limitations in daily activities related to sinonasal symptoms. The RAGE signalling pathway has attracted growing interest in respiratory disease research due to its role in inflammation and oxidative stress. Previous studies have reported inconsistent findings regarding sRAGE in asthma [4,16]. Alzayadneh et al. [16] demonstrated that sRAGE may serve as a potential biomarker of asthma; however, similarly to the authors' results, both sRAGE and FeNO showed limited utility in assessing asthma control in paediatric patients. While some studies reported decreased circulating sRAGE levels in asthmatic children, suggesting a potential protective role through attenuation of airway inflammation, the authors' findings may reflect compensatory upregulation in response to ongoing inflammation or tissue injury. These observations support the hypothesis that distinct inflammatory endotypes may contribute to exacerbation risk in allergic and non-allergic asthma. However, the role of the RAGE axis in paediatric airway disease remains incompletely understood and requires further mechanistic investigation.

An important finding of this study is the demonstration of an association between the severity of nasal and paranasal sinus symptoms and asthma control in the pediatric population. Higher scores on the SN-5 scale were associated with poorer scores on the c-ACT scale. This suggests that children with more severe upper respiratory tract symptoms are more likely to have inadequate asthma control. These findings highlight the functional and immunological link between the upper and lower airways. Inflammatory processes in the nasal mucosa and sinuses may contribute to increased inflammation in the lower airways and to greater asthma instability in children [6,17].

The absence of statistically significant differences in FeNO levels between the groups of children studied, despite varying frequencies of exacerbations, points to the complexity of asthma phenotypes. Fractional exhaled nitric oxide identifies a specific inflammatory endotype associated with type 2 cytokines, in particular interleukin-4 and interleukin-13. This demonstrates that asthma is a heterogeneous disease involving many different inflammatory pathways. Fractional exhaled nitric oxide measurement may not fully reflect the entire spectrum of inflammatory processes leading to loss of asthma control [3,18].

In summary, the results of this study highlight the multi-factorial nature of asthma exacerbations in the pediatric population. The risk of exacerbations depends not only on lower airway function, but also on diseases affecting the upper airways and on novel inflammatory mechanisms identified by biomarkers such as sRAGE.

### Limitations of the study

Several limitations of the present study should be noted. The relatively small sample size may limit statistical power. Second, the cross-sectional design and retrospective assessment of exacerbation history preclude causal inference. Third, biomarkers were measured at a single time point and may have been influenced by recent treatment changes, inhaler adherence, or recovery after exacerbation. Fourth, multivariable analyses were not performed; therefore, potential confounders such as age, sex, treatment intensity, and disease severity could not be fully adjusted for.

Further studies involving larger prospective cohorts are required.

## CONCLUSIONS

The results of this study suggest that asthma exacerbations in children are associated with distinct clinical and biological profiles depending on HDM sensitization status. In children allergic to HDM, more frequent asthma exacerbations were associated with poorer asthma control and lower lung function parameters. In contrast, in children without HDM allergy, exacerbations were associated with higher sRAGE levels and greater sinonasal symptom burden. The study did not show that traditional markers of type 2 inflammation, including FeNO and eosinophil count, allowed for differentiation between children with frequent and infrequent asthma exacerbations. These findings highlight the role of upper respiratory tract symptoms and novel biomarkers of inflammation in identifying children at increased risk of asthma exacerbations. The authors' results may contribute to the development of more personalized approaches to asthma management in the pediatric population. Prospective longitudinal studies are needed to validate these findings and determine their clinical applicability.
